# Exploring the Relationship between G-Quadruplex Nucleic Acids and Plants: From Plant G-Quadruplex Function to Phytochemical G4 Ligands with Pharmaceutic Potential

**DOI:** 10.3390/pharmaceutics14112377

**Published:** 2022-11-04

**Authors:** Andrea P. Falanga, Monica Terracciano, Giorgia Oliviero, Giovanni N. Roviello, Nicola Borbone

**Affiliations:** 1 Department of Pharmacy, University of Naples Federico II, Via Domenico Montesano 49, 80131 Naples, Italy; 2 Department of Molecular Medicine and Medical Biotechnologies, Via Sergio Pansini 5, 80131 Naples, Italy; 3 Institute of Biostructures and Bioimaging, Italian National Council for Research (IBB-CNR), Area di Ricerca site and Headquarters, Via Pietro Castellino 111, 80131 Naples, Italy; 4 Institute of Applied Sciences and Intelligent Systems, Italian National Council of Research (ISASI-CNR), Via Pietro Castellino 111, 80131 Napoli, Italy

**Keywords:** G-quadruplex, G4, tetrads, DNA, RNA, plant, phytochemicals

## Abstract

G-quadruplex (G4) oligonucleotides are higher-order DNA and RNA secondary structures of enormous relevance due to their implication in several biological processes and pathological states in different organisms. Strategies aiming at modulating human G4 structures and their interrelated functions are first-line approaches in modern research aiming at finding new potential anticancer treatments or G4-based aptamers for various biomedical and biotechnological applications. Plants offer a cornucopia of phytocompounds that, in many cases, are effective in binding and modulating the thermal stability of G4s and, on the other hand, contain almost unexplored G4 motifs in their genome that could inspire new biotechnological strategies. Herein, we describe some G4 structures found in plants, summarizing the existing knowledge of their functions and biological role. Moreover, we review some of the most promising G4 ligands isolated from vegetal sources and report on the known relationships between such phytochemicals and G4-mediated biological processes that make them potential leads in the pharmaceutical sector.

## 1. Introduction

**Scheme 1:**
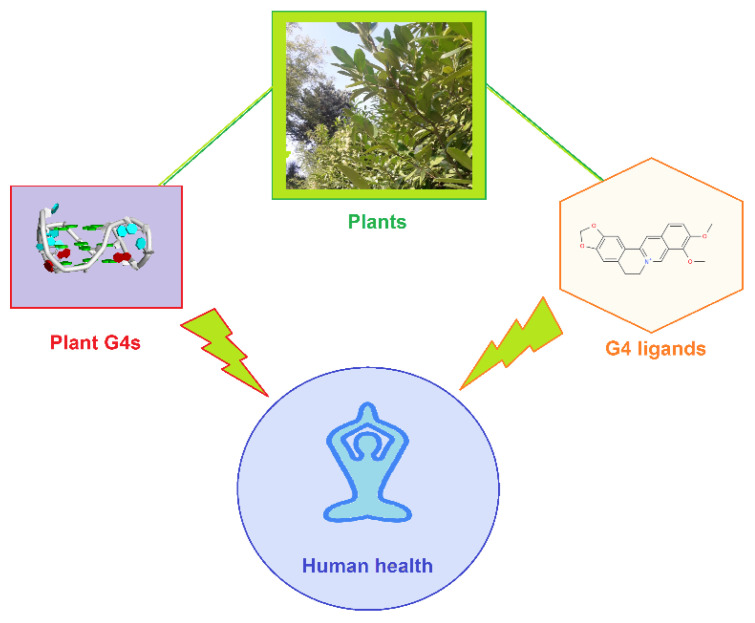
Schematic view of the main goal of the present work: inspiring new perspectives in human disease therapy and diagnostics based on plant G4s and plant-derived G4 ligands (plant photo taken by G.N. Roviello in Naples, Italy—19 October 2022).

Notoriously, in addition to the canonical B-form corresponding to the double-helical nucleic acid, DNA can acquire several other secondary structures. Among these noncanonical structures, G-quadruplex (G4) is an intriguing and deeply investigated four-stranded topology found in both DNA and RNA implicated in several processes in living organisms [1,2,3,4,5,6,7,8,9]. Typically, G4s are built around planar tetrads of guanine nucleobases held together by Hoogsteen H-bonds. G4s constitute an appealing therapeutic target due to their recurrence in the RNA genome of viruses of social relevance, such as SARS-CoV-2 [10,11,12], constituting a druggable target for COVID-19 therapy [13], as well as regulatory regions of the human genome where G4 DNA plays a crucial role in many physiological and disease-related biological mechanisms [14]. Upon G4 function, four-stranded DNA is known to be associated with gene regulation and chromatin remodeling and is also implicated with genetic diseases, genomic instability, and cancer. Therefore, exploring the biological functions of G4 nucleic acids is an appealing research field and is of remarkable significance for innovative therapeutic scenarios concerning human diseases. In particular, G4 DNA regions being found in telomeres and specific sequences of several oncogenes such as c-myc, c-kit, and bcl-2 are considered relevant biomedical targets in anticancer strategies [15,16,17,18,19,20,21,22,23,24,25,26,27,28,29,30]. Interestingly, the promoter region of c-myc—which is over-expressed in most solid tumors and closely related to cancer cell apoptosis, cell-cycle arrest, invasion, proliferation, and metastasis—is known to form a parallel G-quadruplex structure and has been indicated as a valuable target for anticancer strategies [31,32,33,34,35]. Numerous classes of G4 ligands have emerged recently, including the diketopyrrolo[3,4-c]pyrrole derivatives [36], porphyrins, and phthalocyanines [37,38,39]. Remarkably, compounds able to bind and stabilize c-myc G4 led to a downregulation of c-myc expression [40], ultimately determining cancer cell apoptosis with clear advantages in antitumor therapy [41,42,43]. As for the G4 structure, two or more stacks of G-quartets are stabilized by coordination with monovalent cations, typically the ubiquitous potassium or sodium cations, which accommodate in the central G4 channel. Nonetheless, polycations such as positively charged triethylenetetramine and polyamines may contribute to the G4 stability with consequent biological effects [44,45]. Interestingly, oligo-cations, oligo-aromatic binders [46,47,48,49], nucleopeptides [50], and polylysines [51,52] can modify G4 DNA structures as observed, for example, with the human telomeric G4 that is converted by polylysines from the antiparallel to the parallel topology in cations-deficient media [51]. From a more biotechnological perspective, G4-forming guanine-rich nucleic acids may bind proteins or other biomolecules acting as aptamers, which makes them subjects of extensive investigation for their numerous descending biological applications [53,54,55]. Herein we highlight the main G4 functions discovered so far in plants, conscious of their importance in medicinal plant crop improvements. Nonetheless, studying plant non-coding RNA G4s can lead to a better comprehension of G4 regulatory functions that are common among viruses, prokaryotes, protozoa, and humans, ultimately disclosing therapeutic opportunities for a variety of human disorders (Scheme 1) [56].

Another major goal of this work is to give an overview of the main G4 ligands obtained so far from vegetal sources, as they could inspire new efforts to develop convenient G4-targeting strategies making use of plant-derived compounds and their synthetic derivatives (Scheme 1). In fact, natural compounds, due to their polymorphism and structural complexity, often accompanied by low toxicity, may be a very convenient source of anticancer drugs [57,58] and G4 ligands [59], making them attractive assets for G4-targeting drug discovery with potential selectivity for G4 over the more abundant duplex DNA.

## 2. G4 Structures in Plants

Due to the fundamental role of plants in the development of traditional and modern medicine [60,61], and the importance of putative G4-forming regions as a widely conserved set of nucleic sequences that could modulate gene regulation, research on plant G4s holds great relevance in medicinal plant improvement strategies. While the physiological implications of G4 RNA and DNA in plant species have not been much explored yet, their study is of crucial importance for the development of improved pharmaceutical crop varieties for sustainable extraction of therapeutic substances [62].

### 2.1. G4 DNA in Plants

Since G4 DNA formation is believed to be a molecular switch for gene expression, several genome-wide analyses of G4s have been reported for many species [63]. Thousands of potential G4-forming sequences have been identified in guanine-rich (G-rich) regions of eukaryotic telomeric and non-telomeric genomic regions. However, at the beginning of the last decade, only a few putative G4-forming regions were identified in plants. This led Takahashi et al. [64] to develop a two-step strategy to identify G4 motif regions (G4MRs) in plant genomes and classify them on the basis of their positional relationships with the transcription start and termination sites (TSS and TTS, respectively) of plant genes. By using computerized predictive methods, they exhaustively searched for G4 motifs in the whole genome of four plant species, *Arabidopsis thaliana*, *Oryza sativa*, *Populus trichocarpa*, and *Vitis vinifera*. The results unveiled new rules for G4 motif regions in plant genomes and revealed consistent G4MR enrichments in the template strands at TSSs. Overall, the results of this study gave a precious contribution to the elucidation of the functional roles of G4s in plant DNA [64]. The actual or theoretical functions played by G4s in plants were recently reviewed by Griffin B.D. and Bass H.W [65]. Thousands of non-telomeric G4-forming regions have been discovered across several vegetal species, especially near plant gene promoters, suggesting that plant G4s may act as a ubiquitous family of cis-regulatory elements, i.e., non-coding DNA regions that regulate the transcription of neighboring genes. By comparative analyses, it was found that monocotyledons may exhibit up to ten times higher G4-forming region densities than eudicotyledons [65]. Moreover, several studies indicated that the highly diverse plant G4 structures are involved in the regulation of genes implicated in a number of pathophysiological conditions, such as DNA damage and stress response to biotic and abiotic stresses. In other words, more and more studies highlight the emerging functional role of plant G4 motifs in the development of improved crop varieties for sustainable agriculture [62].

Garg et al. identified different types of G4-forming DNA motifs in fifteen sequenced plants and analyzed their distribution in different genomic features, such as coding, promoter, intergenic regions, and gene bodies [66]. G4s with G2-repeats were abundantly detected in all the plant species under investigation. On the other hand, G4s with G3-repeats were linked to intronic, intergenic, and promoter regions, while G2-type G4s were enriched in exonic coding sequences and untranslated regions. Moreover, the study revealed specific sequences present in the conserved genes among monocotyledons and dicotyledons [66]. The same authors found that the genes implicated in development, cell size and growth, transmembrane transport, and gene expression regulation were enriched significantly. Furthermore, they revealed a strong co-occurrence of specific genic motifs with the G4 sequences in the promoter regions and validated the actual formation of G-quadruplexes by G-rich sequences found in several plants. The interaction of G4-forming DNA sequences with plant nuclear proteins was also detected in this study, which overall provided novel insights into the prevalence of G4-forming sequences in plants, demonstrating their association with different genomic aspects and functional evidence [66].

Retrotransposons with long terminal repeats are DNA tracts that form in a significant proportion of eukaryotic genomes. Especially found in plants, they are found in different genes and specific regulatory regions necessary for reverse transcription, and were found to contain several G4-forming sequences as emerged by a recent study conducted on more than 18,000 full-length retrotransposons with long terminal repeats collected from 21 plant species [67]. Specifically, the G4 motifs were often located in the long terminal repeats, both upstream and downstream of promoters, that lead to the whole retrotransposon transcription. Upstream-located G4s were dominant in the negative sense of the DNA strand, while downstream-located G4s prevailed in the positive DNA strand, revealing their role at the level of transcription as well as at the post-transcriptional stages [67]. Using circular dichroism (CD) spectroscopy, it was possible to demonstrate that these G-rich sequences were able to adopt quadruplex structures with different topologies, with some of them being folded as parallel while others as antiparallel G4 structures [67]. Moreover, potential triplex-forming sequences were revealed mainly in the 3′-untranslated region (3′UTR) and, to a lesser extent, in the 5′UTR. Overall, this study revealed a potential role of G4 and triplex DNA as regulator elements of several processes participating in the life cycle of long terminal repeat-containing retrotransposons and as potential recombination sites during the genome rearrangements based on retrotransposons [67]. Bioinformatic studies were also conducted to explore the biological pathways involving G4 structures in plants [68] that were previously studied more in the context of human diseases than that of vegetal organisms. In particular, the bioinformatic investigation of Volná et al. was accompanied by a complex CD spectroscopic study aimed at identifying stable G-quadruplexes in the gene RPB1, conserved across different plant and mammal species, which codes for the large subunit of the RNA polymerase II. It was shown that the RPB1 G4-forming locus is highly evolutionarily conserved among plants belonging to the *Archaeplastida* kingdom, sharing a common ancestor older than one billion years. The described plant G4s were also hypothesized to interact with UV light, potentially leading to an additional layer of the regulatory network [68].

Telomeres are structures consisting of repeats of the short G-rich sequence TTAGGG found in eukaryotes and especially studied in mammalians [69]. Located at the ends of linear chromosomes, they play fundamental roles in the context of genomic stability. While it is notorious that the mammalian telomeric G-rich repeats are able to form G4 structures able to modulate telomere functions [69], less studied are DNA telomeres in plants. These show TTTAGGG (in place of TTAGGG) DNA sequence repeats which play an essential role in plant growth and development, as well as in environmental adaptation [70]. Plant telomeric G4 structures were identified through bulk and single-molecule assays, including single-molecule FRET approaches and CD spectroscopy, that led to the complete characterization of the dynamics and the structure of the plant telomeric DNA sequence GGG(TTTAGGG)_3_. This typical telomeric sequence was able to fold into mixed G4 structures, including parallel and antiparallel topologies, in the presence of potassium cations. Intermediate dynamic transitions, including G-based hairpin, parallel triplex, and antiparallel triplex structures, were also detected. Interestingly, the model telomeric G4 structure was unfolded by AtRecQ2 helicase but left untouched by AtRecQ3 [70]. Another study aiming at highlighting the functional relevance of plant G4s in evolutionarily distinct plant species analyzed the genome of garden pea (*Pisum sativum*), a unique member of the *Fabaceae* family, showing that it contains several putative G4-forming DNA sequences [71]. Intriguingly, these G4 motifs were located nonrandomly in the nuclear genome of garden peas. Remarkably, other putative G4-forming sequences were found in chloroplast and mitochondrial DNA, and the G4 structure formation was experimentally confirmed for the sequences found in both organelles. The frequency of putative G4-forming sequences for nuclear DNA was in the same range as for chloroplast DNA (ca. 0.5/kbp) but significantly lower when compared to mitochondrial DNA (1.6/kbp) [71]. While putative G4-forming structures found in the nuclear genome were associated mainly with regulatory regions, including 5′UTRs, as well as upstream of the ribosomal RNA region, they were located around RNA genes in mitochondrial DNA and chloroplast DNA. The non-random localization of putative G4-forming sequences uncovered their functional and evolutionary significance in the garden pea genome [71]. Using bioinformatics techniques, Wang et al. explored different putative G4 motifs in several genomic regions of *Oryza sativa*, in particular studying two subspecies (*indica* and *japonica*) and the whole genome of eight other plant species [72]. After this analysis was performed on all ten plant species, they found G4 motif density in monocotyledons higher than in dicotyledons. A wide distribution of putative G4-forming DNA sequences was found in the *O*. *sativa* genome. The G4 motifs were more abundantly located into 5′UTR and near transcription start sites with relatively high enrichment [72] leading to the hypothesis that G4 in the plant species investigated was involved in gene transcription and consequent translation. Moreover, analyzing the distribution of different loop lengths in G4, the same authors estimated the density of putative G4-forming sequences in the long loop that was lower than the short loop in the intron of *indica* subspecies, while it did not differ significantly from that found in *japonica*. In addition, focusing their attention on the loci with putative G4-forming sequences and conducting gene ontology analysis of them, Wang et al. identified several gene ontology terms that were highly correlated with the loci containing at least one G4 motif. The gene ontology analysis in the two subspecies of *Oryza sativa* furnished a useful example for elucidating the functional roles of G4 in plants [72].

### 2.2. G4 RNA in Plants

Plants contain several non-coding RNA G4 structures endowed with regulatory functions common also to viruses, prokaryotes, protozoa, and humans [56]. On the other hand, Yang et al. identified several RNA sequences rich in guanine in the plant transcriptome, whose folding potential was profiled in vitro and which were revealed to be potentially able to form G4 structures [73]. More in detail, using both high-throughput sequencing and cell imaging methods, the same authors detected RNA G4s at the genome-wide scale as well in living cells [74]. A global abundance of RNA G4 motifs with two G-quartets was observed, with the global RNA folding potential being highly influenced by these four-stranded secondary structures. Remarkably, both in vitro and in vivo RNA chemical structure profiling techniques revealed hundreds of RNA G4 structures strongly folded in both mouse-ear cress (*Arabidopsis thaliana*) and rice (*Oryza sativa*) and furnished for the first time direct evidence of the formation of RNA G4 structures in living eukaryotic cells [73]. Furthermore, biochemical and genetic analyses indicated that RNA G4 folding regulates the translation process, ultimately modulating plant growth. Overall, Yang et al. not only demonstrated for the first time the existence of RNA G4s in vivo but also indicated that RNA G4 structures play different and often not sufficiently explored roles in the regulation of plant growth and development [73]. Not less importantly, recent investigations have also emphasized the central role that RNA structures play in plant adaptation. More specifically, among the several highly complex structures of RNA, G4s widespread across the transcriptomes of a number of plant species, as evidenced by several computational predictions and also experimentally demonstrated [74], are regulatory motifs in vegetal organisms important for their adaptation to the most diverse environmental factors along with evolutionary perspectives [74]. Aiming at investigating the role of nucleotide composition in determining gene functionality and the ecological adaptation of plant species to distinct environmental conditions and the underlying biological function of nucleotide composition determining the environmental adaptations, Yang et al. recently systematically studied the nucleotide compositions of transcriptomes across 1000 plants and their corresponding habitats [75]. Interestingly, it emerged that plants growing in cold climates have G-enriched transcriptomes, which can readily fold into RNA G4 structures. By immunofluorescence detection and in vivo structure profiling studies they found that RNA G4 structure formation in plants was significantly enhanced in response to cold [75]. Cold-responsive RNA G4 structures were found to strongly enhance the stability of mRNA, rather than affecting its translation. Conversely, disrupting the RNA led to mRNA decay in the cold, and impaired responses of the plant to the cold. The results of this study suggested therefore that evolutionarily plants adopted RNA G4 structure as a molecular marker to improve their adaptation to the cold [75]. The folding of fragments of transfer RNA (tRNA) into G4 structures and the implications of G4 in translational inhibition have been studied in plants and compared with mammalian systems. In particular, the influence of human and plant fragments of tRNA and model G4 structures on translation in wheat germ extract and rabbit reticulocyte lysate was demonstrated by Jackowiak et al. [76], who were able to associate the efficiency of translational inhibition in the mammalian system with the type of G4 topology. However, the same authors observed that in plants, the ability of a small RNA to adopt the G4 structure was not sufficient to block the translation process, suggesting that other structural determinants are implicated in this feature [76]. In the context of the exploration of G4 structure formation and the consequent biological role in plants, an experimental study based on CD titration, UV melting, in-line probing and reporter gene assay studies led to the discovery of a plant RNA G4 structure that was able to inhibit the RNA translation in *Arabidopsis thaliana* [77]. Such a G4 motif was located within the 5′UTR of the mRNA and the G4 structure was found to be highly stable and thermodynamically favored over a competing hairpin structure in the 5′UTR at physiological potassium and magnesium concentrations. Transient reporter gene assays conducted in living plants showed that the G4 structure inhibited the translation but not the transcription process, indicating this G4 structure as a translational repressor in vivo. Moreover, the in-line probing assay led to the elucidation of the secondary structure of the RNA supporting the formation in vitro of the G4 structure in the context of the complete 5′UTR [77].

### 2.3. Plants G4 Binding Proteins

Aware of the importance of proteins able to bind G4 structures [78,79] as valuable targets for strategies aiming at modulating G4-related processes in different organisms, Volná et al. also investigated them bioinformatically in plants. G4 binding proteins were screened, inspecting the available plant protein sequences in order to detect the best protein candidates with the potential to bind G4 structures [80]. The authors started from the consideration that two similar arginine and glycine-rich G4-binding motifs were previously reported in humans: the so-called “RGG motif” (with the amino acid sequence RRGDGRRRGGGGRGQGGRGRGGGFKG), and the more recently described “NIQI motif” (whose sequence is RGRGRGRGGGSGGSGGRGRG). With this information in mind, they screened plant proteins that included the abovementioned motifs in their amino acid sequences using two bioinformatic approaches (BLASTp and FIMO scanning) [80]. They found numerous proteins containing the G4-binding motifs in common with humans and were able to describe the core proteins involved in G4 folding and resolving in algae and green plants, including *Arabidopsis thaliana*, the plant model organism of their study. The emerged G4-binding protein candidates were sorted by their physiological and molecular functions and were hypothesized to play significant roles in the regulation of gene expression in plants [80].

## 3. Phytochemical Compounds as G4 Ligands

**Scheme 2:**
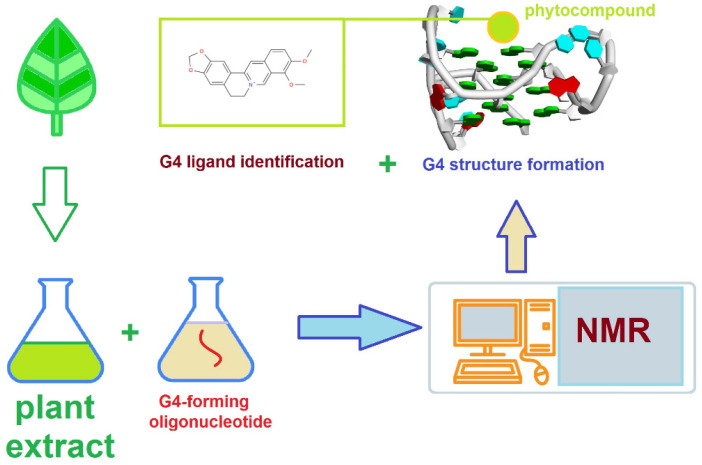
Schematic representation of the strategy adopted by Zhou et al. [82] for G4-ligand identification from plant extracts.

Searching for new lead compounds from plant extracts constitutes a promising strategy to find effective drugs characterized by low toxicity and conveniently obtainable in high amounts from natural sources. In this context, the screening of G4 ligands from vegetal sources is particularly important because these phytochemicals may act as potential antitumor drugs. This is due to the previously discussed implication of G4-forming sequences in cancer as recurrent elements in the promoter region of various oncogenes. This designates these G-rich motifs as key targets for anticancer binding agents, and as motifs involved in the maintenance of human telomeres, since the stabilization of telomeric DNA in G4 structures has the potential to evolve as a novel anticancer therapy [81]. A simple method for the fast identification of G4-binding compounds from plant extracts was proposed by Zhou et al. (Scheme 2) who took advantage of the detection of specific signals diagnostic for the presence of G4s in solution by ^1^H NMR spectroscopy [82].

**Figure 1:**
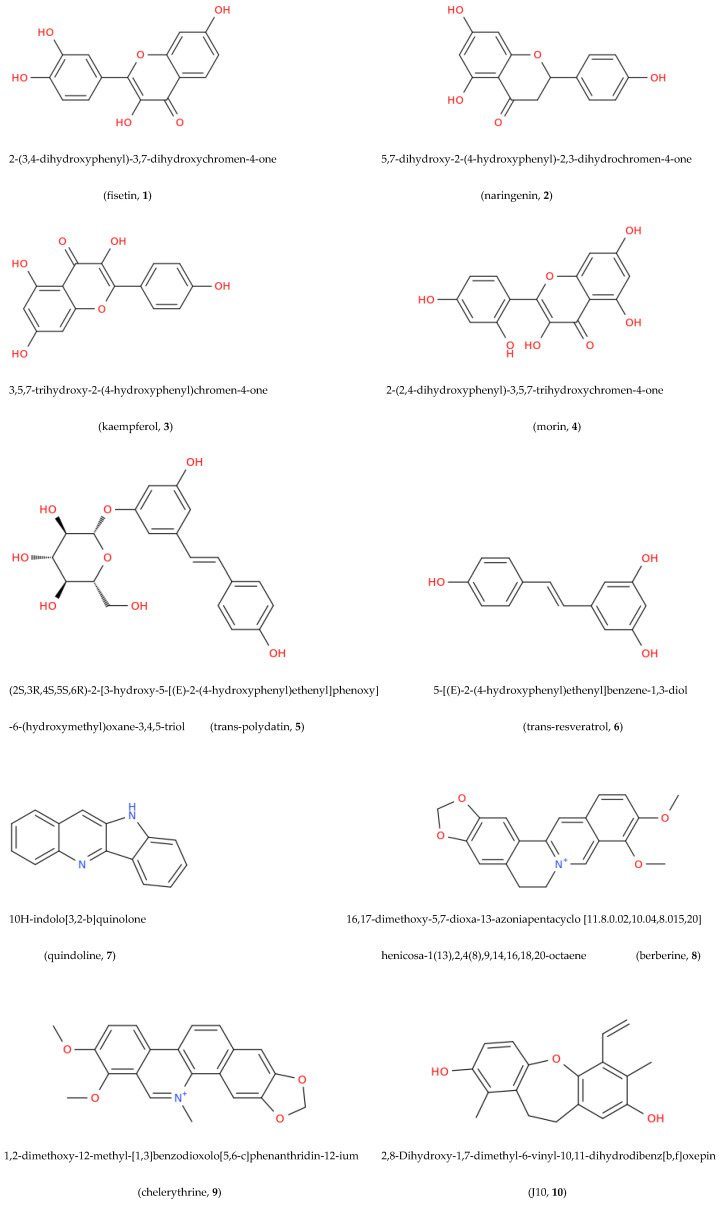
Structural representation of some of the phytochemical G4 ligands mentioned in this work, with IUPAC and common names.

Further, they exploited the NMR spectral shift of the G4-diagnostic imino proton signals (*δ* 10–12 ppm) following the incubation with potential G4 ligands as an in vitro bioassay to reveal the existence or absence of G4 ligands [82]. At the same time, the structure of the phytochemical ligands was readily identified without the need for any prior chromatographic separation by coupling the fast G4 recognition method with the efficiency of NMR-based structure elucidation, which led to the successful screening of a G4 phytochemical ligand within a simulated plant extract [82]. In a different study, Shang et al. used a combination of dialysis and NMR screening to successfully fish out G4 ligands from the aqueous ethanolic extract of *Phellodendron chinense Schneid* cortexes [83]. Within the several classes of flavonoids endowed with health-promoting properties, including those with anticancer potential, flavonols are among the most promising sources of new therapeutics. Aiming at facilitating the discovery of highly selective G4 binders based on the flavonol scaffold, the ability of two structurally related dietary flavonols isolated from plants, fisetin (**1**, Figure 1) and naringenin (**2**), to bind G4s was investigated in comparison with the double-stranded DNA [84]. The main feature that emerged from this study was that even small structural differences in closely related chemical classes of small molecules can lead to terrific effects in their binding affinity with two different secondary structures of DNA.

In fact, taking advantage of different modeling and spectroscopic methods, it was possible to demonstrate a differential binding ability of the two flavonoids toward G4 and double-helical DNA targets [84]. Phytochemical **1** is more strongly bound to parallel G4 than the double-helical DNA structure, while **2** showed higher binding affinity to the double-helical rather than the four-stranded DNA target. Computational approaches based on molecular docking results confirmed the trend observed by the spectroscopic techniques and remarkably suggested that both phytochemical ligands were stacked externally in the G4 structure. This led to the conclusion that ring planarity of the ligand structure appeared to be a crucial factor for the preferential G4 over the double-helical DNA recognition of flavonoids [84].

Moreover, the ability of the plant flavonols kaempferol (**3**, Figure 1) and morin (**4**) to interact with different G4 sequences as well as double-helical DNA was assayed through a combined strategy making use of both computational (molecular docking) and experimental (spectroscopic) studies. In this respect, CD spectroscopy is a spectroscopic technique typically employed to verify the formation of several secondary structures of natural oligonucleotides and DNA analogs [85,86,87], including the G4 structures in G-rich sequences [88,89,90,91,92]. Importantly, CD spectroscopy can give information on the ability of potential DNA binders to alter the denaturing temperature of a DNA secondary structure acting as a stabilizer or destabilizer [93]. Interestingly, the phytocompound **3** preferentially bound to VEGF G4 DNA compared to other G4 sequences and the reference double-helical DNA target [94]. In addition, the interaction of **3** with the VEGF G4 increased the thermal stability of the latter, disclosing the potential use of this flavonol in the gene regulation of cancer cells. Conversely, **4** showed a significantly weaker interaction ability toward both G4 (various models) and double-helical DNAs, showing no appreciable specificity in binding different DNA structures [93]. The different behavior of **3** and **4** in DNA binding was associated with an important role of the 2′-OH moiety in the B-ring of the flavonol moiety. While **4** adopts an essentially nonplanar conformation due to steric hindrance from the additional 2′-OH substituent, **3** is almost planar and this structural difference has a key role in differentiating the two plant compounds with respect to their ability to interact with VEGF G4 and other DNAs. [94]. Recently, the G4 binding properties of trans-polydatin (**5**, Figure 1), the 3-β-D-glucoside form of the polyphenol trans-resveratrol (**6**) [95], a plant-derived compound known for its numerous therapeutic effects [96,97], were explored using three cancer-related G-rich DNA targets, including a G-rich region found in the promoter of the c-myc oncogene. These investigations based on both computational [98] and experimental methods [99] revealed that **5** is able to bind all of the screened G4s, displaying a capability to discriminate G4 over double-helical DNAs higher than **6** and inhibit melanoma cancer cell proliferation [99]. Mechanistically, the anticancer effects of **5** on the inhibition of cell proliferation and metastasis were shown to occur through the suppression of the c-myc expression, as proven in studies using models of human cervical cancer [100]. Structurally related to cryptolepine [101], quindoline (**7**, Figure 1) is another naturally occurring alkaloid extracted from the African plant *Cryptolepis sanguinolenta* traditionally used as antimalarial herbal remedy [102]. This phytochemical and its derivatives were found to bind the human telomeric G4s, displaying modest cytotoxicity against several cancer cell lines as well as inhibitory activity against the telomerase enzyme, with IC_50_ values in the 6–16 μM range [103].

**Figure 2:**
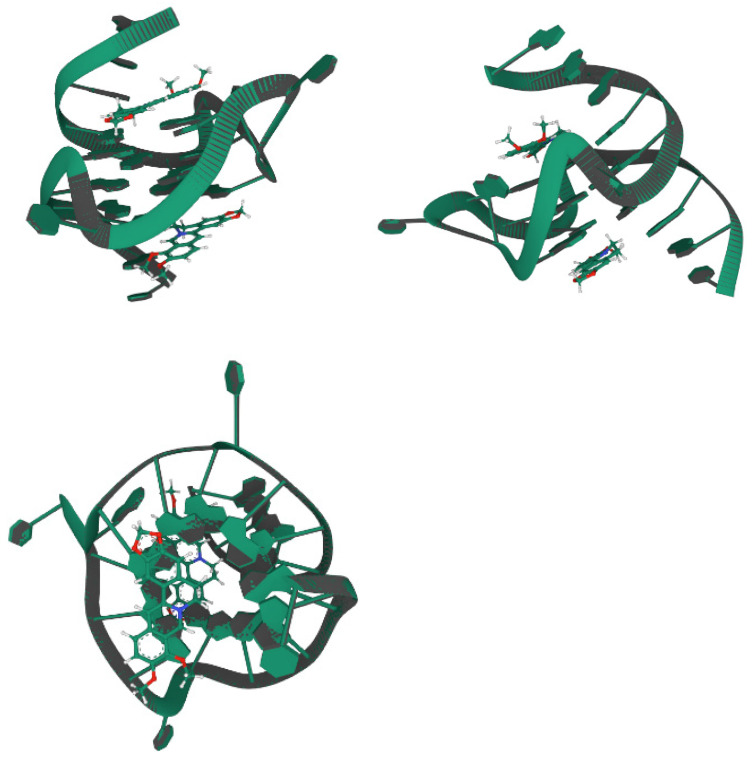
Three-dimensional views of the complex formed between c-myc G4 DNA and **8** (PDB ID: 7N7E, images elaborated from https://www.rcsb.org/3d-sequence/7N7E?assemblyId=0, accessed on 23 September 2022) [104].

The plant alkaloid berberine [104] (**8**, Figure 1) isolated from several Chinese herbs and known for its antimicrobial properties, has gained increasing attention also for its anticancer effects being able to suppress cell growth in a variety of cancers including prostate carcinoma, breast cancer, and gastric carcinoma, to cite only a few [105]. Remarkably, **8** emerged from a rapid screening of G4 ligands from plant extracts achieved using a combined approach based on diffusion-ordered ^1^H NMR spectroscopy (DOSY) and 2D NMR techniques [106]. This strategy was endowed with a low detection limit and led to the identification of berberine as a G4 ligand in two different plant extracts and its structural elucidation without the need for any prior in vitro binding or functional assays. As for the detection limit of this method, it was estimated to be around 0.06% mass concentration of G4 ligand in the plant extracts [106]. Interestingly, **8** and especially its 9-*O*-substituted derivatives bearing side chains with terminal amino groups were able to stabilize telomeric G4s [105]. Structurally, the binding of **8** with telomeric G4 was described by X-ray that showed two units of **8** simultaneously interacting with each external G-tetrad via π-stacking, while the crescent-shaped ring of **8** was seen stacked over two guanines [105,107]. The structure of the complex of **8** with the parallel c-myc G4 was also described in solution by Dickerhoff et al. through NMR analysis (Figure 2).

It was shown that this plant alkaloid binds the G4 DNA using a base-recruitment mechanism with a reversed orientation, with the positively charged convex side of the alkaloid positioned above the G4 tetrad center [104]. Other G4 binding studies were conducted on the plant alkaloid chelerythrine (**9**, Figure 1), a tetracyclic aromatic compound isolated from *Macleaya cordata* belonging to the family of benzo[c]phenanthridines and endowed with amyloid aggregation modulatory properties [108]. Remarkably, **9** was explored as a potential anticancer agent and was demonstrated to be capable of inhibiting the human telomerase activity via the stabilization of telomeric G4 DNA. The structural characterization of the resulting **9**/G4 DNA complexes and the estimation of the chelerythrin-induced G4 thermal stabilization were achieved using optical (UV and CD) and NMR spectroscopies, and calorimetric techniques (including differential scanning calorimetry (DSC) and isothermal titration calorimetry), respectively [109]. The stoichiometry of the most stable 9/G4 complex was found to be 2:1, while the thermal stabilization induced by **9** was estimated to be around 24 °C. NMR results revealed the ability of **9** to interact with both the G4 tetrads and the phosphate backbone. Molecular dynamics studies indicated a significant binding specificity of **9** toward G-quartets over groove binding. According to this model, out of the two ligand molecules involved in the complex with the telomeric G4 DNA, the first chelerythrin molecule binds one of the external G-quartet followed by a second molecule which interacts with the G4 groove, possibly leading to the aggregation of the G4 DNA which was experimentally observed by NMR [109]. Moreover, the binding of **9** with the G4 formed by the c-myc Pu27 sequence was investigated by molecular docking and molecular dynamics approaches [110]. This study revealed that **9** stacks over the 5′ end of the c-myc G4 DNA sequence and that the methoxylation at position 12 favors the binding, enhancing the van der Waals energy contribution, suggesting further synthetic modifications to the skeleton of **9** to improve the anticancer activity. The binding of **9** to the same G4 target was also studied experimentally using calorimetry. When added to a prefolded c-myc G4 structure, **9** formed a 2:1 complex which was more thermally stable than the free G4 DNA. It was demonstrated that the ability of **9** to prevent the hybridization of Pu27 with its complementary DNA strand is due to the stabilization of the Pu27 G4 structure [111]. Overall, this work led to the hypothesis that the anticancer activity of **9** may be the result of its combined effects on telomeres maintenance and the downregulation of the c-myc oncogene. By screening crude extracts from 17 medicinal plants for G4 binding activity through ^1^H NMR spectroscopy, it was found that *Peganum harmala* L. is a source of several G4 ligands all belonging to the family of pegaharmines, some of which are selective binders of biologically relevant parallel DNA G4s, such as those formed in the c-myc, bcl-2 and VEGF promoters, with respect to (3 + 1) hybrid G4s, such as those formed in telomeric DNA [112]. Finally, several phytocompounds isolated from Juncaceae, including benzocoumarin, dihydrophenanthrene, and dihydrodibenzoxepin derivatives, were screened as G4 binders by affinity chromatography-based assays, and the compound J10 (**10**, Figure, [113] Figure 1), endowed with the highest affinity and selectivity for G4 DNA, underwent further fluorescence spectroscopy and CD characterization [114]. The results of this experimental study associated with molecular docking suggested the capability of **10** to selectively bind to cancer-related G4s over double-helical DNA due to its ability to bind at the grooves of telomeric and oncogenic G4s, also proving the thermal stabilizing effect of **10** as well as a certain binding preference for the parallel G4 topology. Biological assays showed that **10** exerted antiproliferative effects on human leukemia cells by activating the apoptotic pathway, with no significant effects on healthy human fibroblasts [114].

## 4. Conclusions

Plants are sources of a cornucopia of bioactive phytochemicals with potential utility in the prevention and treatment of diseases. Several plant-derived G-quadruplex ligands have been described in the last decades. Given their polymorphism, structural complexity, and selectivity for G4 over other DNA secondary structures, these phytochemicals may be valuable assets for G4 targeting. Overall, plant G4 ligands could inspire future efforts to develop valuable G4-targeting therapeutic strategies using both natural compounds and their ad hoc-designed synthetic derivatives. Concluding this review we would like to underline the importance of the transition from plant genome to human health [115]. In this regard, the study of plant G4s regulating the expression of genes involved in pathophysiological conditions, including plant responses to biotic and abiotic stresses, is expected to disclose novel scenarios in improving medicinal crops as convenient sources of pharmaceutical compounds with the potential to improve human health. In fact, many plant-derived therapeutics are currently produced in very small amounts or plant species of a not-easy culture. Therefore, a deeper knowledge of plant genomics, and particularly the plant G4 role, will facilitate our ability to produce pharmaceuticals and nutraceuticals in convenient and sustainable fortified plant systems, even under difficult cultivation environments [116]. In addition, given the common regulatory functions of non-coding G4-forming RNA in several organisms, including protozoa, prokaryotes, plants, and humans, and considering the potential biomedical role of strategies aiming at stabilizing/destabilizing such RNA G4s in humans, the study of plant G4 RNAs and their interactomes could inspire new therapeutic opportunities for many human pathologies, from cancer to infectious and neurodegenerative diseases.

## Data Availability

Not applicable.
